# Point-of-Care Ultrasound Detection of a Right Atrial Thrombus Leading to AngioVac Thrombectomy in a Patient With Sickle Cell Crisis

**DOI:** 10.7759/cureus.107762

**Published:** 2026-04-26

**Authors:** Mateen Sheikh, Adriana Medina, Marina Shehata, David Casto, Guillermo Izquierdo-Pretel

**Affiliations:** 1 Internal Medicine, Florida International University, Herbert Wertheim College of Medicine, Miami, USA; 2 Hospital Medicine, Jackson Memorial Hospital, Miami, USA

**Keywords:** angiovac system, catheter-related right atrial thrombosis, central venous catheter (cvc), point-of-care ultrasound (pocus), sickle-cell disease, vaso occlusive crisis

## Abstract

Point-of-care ultrasound (POCUS) has emerged as a valuable extension of the bedside physical examination, enabling rapid identification of cardiac abnormalities that may not be clinically apparent. Intracardiac masses, particularly in the right atrium, represent a diagnostic challenge and carry significant clinical implications, including the risk of embolization. Early recognition is critical to guide timely management.

We report the case of a patient admitted with vaso-occlusive sickle cell crisis who underwent bedside cardiac ultrasound as part of the clinical evaluation. POCUS revealed a previously undocumented, mobile right atrial mass measuring approximately 3.1×2.2 cm near the junction of the inferior vena cava and right atrium, not associated with the tricuspid valve and without prolapse into the right ventricle. A prior transthoracic echocardiogram done six months ago had not reported this finding. Subsequent formal echocardiographic evaluation confirmed the presence of a right atrial mass concerning for thrombus. Given the size, mobility, and potential embolic risk, the patient underwent mechanical thrombectomy using the AngioVac system, with successful removal of a significant thrombotic burden.

Right atrial thrombi are uncommon but clinically significant due to their association with thromboembolic events. This case highlights the role of POCUS in the early detection of intracardiac pathology and its impact on clinical decision-making. Prompt identification at the bedside facilitated expedited diagnostic confirmation and definitive intervention.

POCUS can serve as a critical diagnostic tool in identifying unexpected intracardiac abnormalities, enabling early intervention in high-risk conditions. Its integration into routine hospital medicine practice may facilitate early detection of high-risk intracardiac thrombi and enable timely intervention.

## Introduction

Right atrial thrombi (RAT) are rare but potentially life-threatening entities due to their association with pulmonary embolism, hemodynamic compromise, and increased mortality. They most commonly occur in the setting of indwelling central venous catheters, hypercoagulable states, or underlying cardiac pathology. Patients with sickle cell disease represent a uniquely high-risk population, given the interplay of chronic inflammation, endothelial dysfunction, and a well-established hypercoagulable state, all of which predispose to thrombus formation [1,2]. RAT have been classified as Type A, free-floating thrombi in transit; Type B, in situ thrombi attached to the atrial wall or intracardiac devices; and Type C, rare mobile thrombi that may resemble an atrial myxoma. Despite this elevated risk, intracardiac thrombi are often underrecognized and typically identified only when advanced imaging is performed for specific clinical indications.

Point-of-care ultrasound (POCUS) has emerged as an important extension of the bedside physical examination, enabling rapid identification of clinically significant findings. Although its utility is well established in the assessment of volume status, pericardial effusion, and gross ventricular function, its role in the incidental detection of intracardiac thrombi in high-risk populations is not well characterized in the literature [3]. We present the case of a patient admitted with vaso-occlusive sickle cell crisis in whom bedside cardiac POCUS, performed for unrelated clinical indications, revealed a previously undocumented large right atrial thrombus associated with an indwelling central venous catheter. This finding prompted confirmatory imaging and expedited percutaneous mechanical thrombectomy with the AngioVac system, illustrating how bedside POCUS can identify unsuspected high‑risk intracardiac pathology and directly alter patient management [4,5].

## Case presentation

Clinical history

A 32-year-old woman with sickle cell disease, iron overload from chronic transfusions, bilateral hip avascular necrosis status post bilateral hip arthroplasty, and prior acute chest syndrome was admitted to a tertiary hospital in Miami for the management of an acute vaso-occlusive crisis in the setting of newly diagnosed early pregnancy. On presentation, she was hemodynamically stable: temperature at 36.9°C, heart rate at 106 bpm, respiratory rate at 18/min, blood pressure at 126/79 mmHg, oxygen saturation (SpO_2_) of 100% on room air, with laboratory findings consistent with hemolysis, including anemia with hemoglobin at 8.7 g/dL, elevated reticulocyte count at 7.5%, leukocytosis at 21.9 ×10^3/µL, mild indirect hyperbilirubinemia (1.9 mg/dL), and bicarbonate 19 mEq/L, without evidence of acute infection, organ dysfunction, or myocardial injury (troponin 0.01 ng/mL).

**Figure 1:**
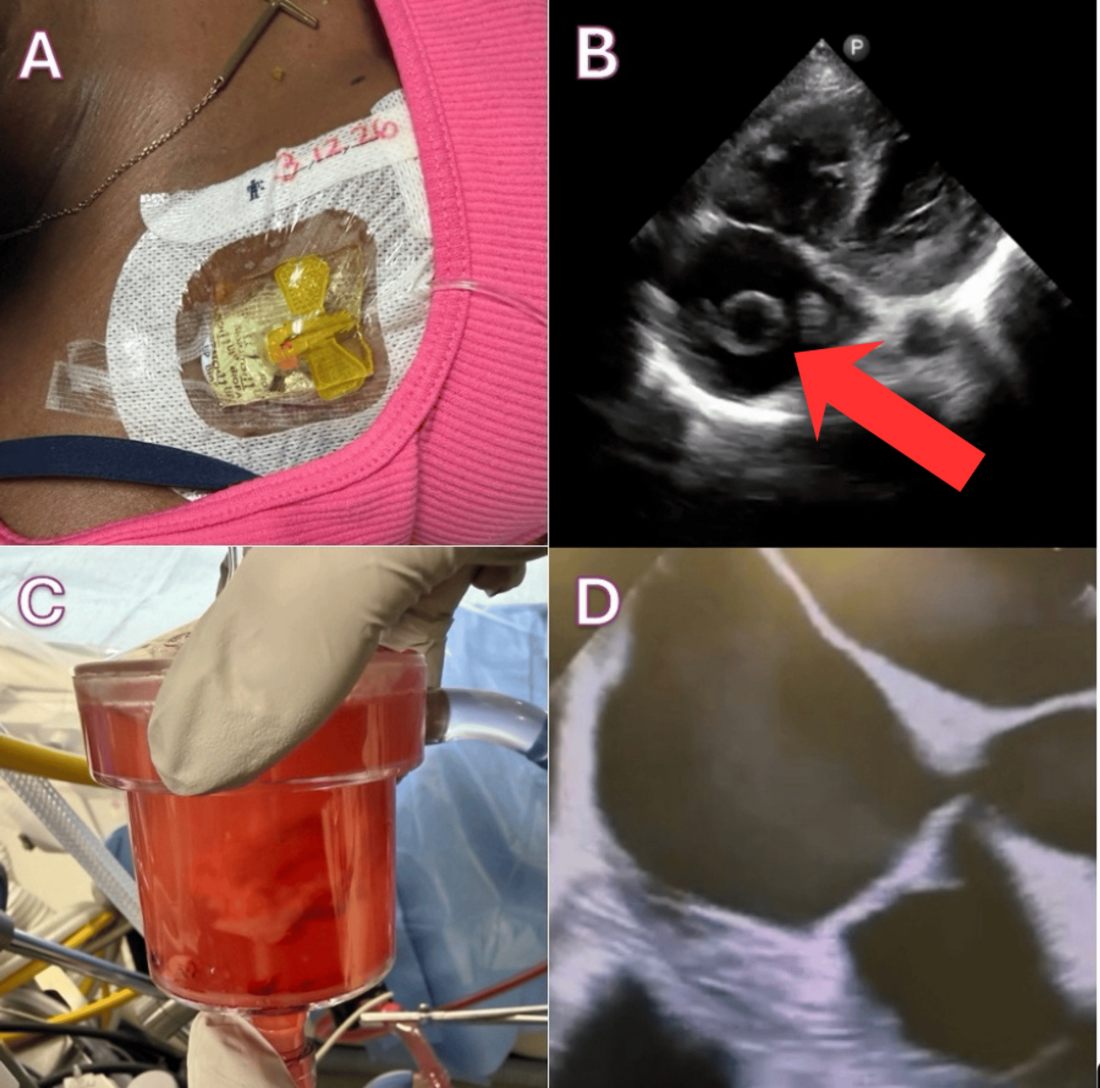
(A) Implanted venous access port present on right chest. (B) Right atrial mass on four apical chamber view, present on bedside POCUS, and later found to measure 3.1×2.2 cm on formal transthoracic echocardiography. (C) Thrombotic material retrieved during AngioVac thrombectomy, demonstrating a large burden of intracardiac thrombus consistent with prior echocardiographic findings of a right atrial mass. (D) Right atrium free of thrombus as shown on intraoperative transesophageal echocardiography. POCUS: Point-of-care ultrasound.

Initial obstetric evaluation demonstrated a pregnancy of unknown location, with subsequent imaging confirming an early intrauterine pregnancy. Her vaso-occlusive pain was managed with opioid therapy through her implanted central venous access port, with gradual improvement during hospitalization (Figure 1A). She had no fever, nausea, vomiting, or abdominal pain. The review of systems was otherwise unremarkable, including no shortness of breath, palpitations, or edema.

Although imaging six months prior had not documented catheter-related complications, subsequent echocardiographic evaluation suggested that the catheter tip was positioned within or near the right atrium, raising concern for catheter-associated thrombus formation in the setting of an underlying hypercoagulable state. On physical examination, she had diffuse bony tenderness over the hips and long bones, no jugular venous distension, clear lungs on auscultation, regular heart rhythm without murmurs, and no peripheral edema. Abdominal exam was benign.

During admission, the patient reported mild upper respiratory symptoms, prompting bedside POCUS to evaluate for pulmonary pathology. During this evaluation, an incidental right atrial mass was identified, prompting further cardiac workup and multidisciplinary consultation.

Diagnostic evaluation

Initial bedside cardiac POCUS using a Philips Lumify portable ultrasound system (S4-1 phased array transducer; Philips Healthcare, Bothell, WA, USA) equipped with a phased array transducer (S4-1), optimized for cardiac imaging, revealed a large, mobile echogenic mass within the right atrium. The differential diagnosis for a right atrial mass includes thrombus, vegetation, benign or malignant cardiac tumor, and artifact; however, thrombus was favored given the patient’s indwelling central venous port, hypercoagulable state from sickle cell disease and pregnancy, and the mobile appearance of the mass and its location near the inferior vena cava-right atrial junction without valvular attachment or prolapse into the right ventricle. This prompted further evaluation with formal transthoracic echocardiography, which confirmed a ~3×2.5 cm right atrial mass consistent with thrombus (Figure 1B and Video 1).

**Video 1:** Right atrial mass on four apical chamber view, present on bedside POCUS

Therapeutic intervention and follow-up

In this case, given the large size and mobility of the thrombus and the associated embolic risk, percutaneous mechanical thrombectomy using the AngioVac system (AngioDynamics, Latham, NY, USA) was pursued. This minimally invasive approach allows for the removal of thrombotic material while avoiding the risks associated with open cardiac surgery or systemic thrombolysis (Figure 1C, 1D). 

Following thrombectomy, repeat imaging demonstrated successful removal of the right atrial thrombus with no residual mass. The patient remained hemodynamically stable and experienced no procedural complications. Her vaso-occlusive pain improved, allowing transition to oral analgesics prior to discharge.

She was discharged with close outpatient follow-up and appropriate management of anticoagulation. This case highlights a favorable outcome in which an incidentally detected intracardiac thrombus was promptly identified and definitively treated, underscoring the clinical impact of bedside POCUS in detecting otherwise unsuspected, high-risk pathology.

## Discussion

Right atrial thrombi may be clinically silent or present with nonspecific symptoms, contributing to delayed diagnosis. In this case, the patient’s presenting symptoms were unrelated to intracardiac pathology, and imaging six months prior had not identified the thrombus, raising considerations regarding either rapid thrombus formation or limitations of earlier studies. Based on its location near the inferior vena cava-right atrial junction and the context of an indwelling central venous catheter, the lesion was most consistent with a Type B right atrial thrombus. Additional imaging modalities, such as transesophageal echocardiography or CT angiography, may be useful in similar cases to further characterize thrombus morphology, attachment, and embolic risk.

The diagnosis of catheter‑associated right atrial thrombus was established based on imaging findings in the context of an indwelling central venous catheter with the tip positioned in or near the right atrium, combined with the underlying hypercoagulable state associated with sickle cell disease. These thrombi carry a significant risk of embolization, particularly when mobile, with reported mortality varying according to thrombus characteristics and management strategy, underscoring the importance of early detection and timely intervention [6,7].

Management options for right atrial thrombi include systemic anticoagulation, thrombolytic therapy, surgical thrombectomy, and percutaneous mechanical thrombectomy. The choice of therapy depends on thrombus size, mobility, patient stability, and embolic risk [7]. In this case, AngioVac thrombectomy was favored because the thrombus was large, highly mobile, and potentially embolic, making prompt mechanical removal preferable to anticoagulation alone, which would not provide immediate clot resolution. Systemic thrombolysis was less desirable because of bleeding concerns in the setting of early pregnancy and the uncertainty surrounding a large, mobile right atrial mass, while surgical thrombectomy would have been more invasive. Emerging evidence supports the safety and efficacy of percutaneous mechanical thrombectomy systems such as AngioVac as a less invasive alternative in appropriately selected patients [4,5].

This case underscores the utility of POCUS as a rapid bedside diagnostic tool capable of identifying clinically significant and otherwise unsuspected intracardiac pathology. Although not traditionally emphasized for this purpose, cardiac POCUS can detect large intracardiac thrombi when image acquisition and interpretation are performed systematically [3]. These findings support incorporating focused cardiac views during non‑cardiac POCUS examinations in high‑risk patients.

Furthermore, this case reinforces the thrombotic risk associated with indwelling central venous catheters, particularly in hypercoagulable conditions such as sickle cell disease. Catheter‑associated right atrial thrombi are well described but likely underdiagnosed, especially in patients without overt cardiopulmonary symptoms. Early detection is critical given the potential for serious complications, including pulmonary embolism.

Given the elevated thrombotic risk in patients with sickle cell disease, particularly in the presence of indwelling central venous catheters, this case raises the question of whether targeted incorporation of focused cardiac POCUS may be beneficial in selected high‑risk populations. Although current evidence does not support routine screening for intracardiac thrombi, selective use of bedside ultrasound in patients with persistent catheters and hypercoagulable states may facilitate earlier detection of clinically silent thrombi. Further studies are needed to evaluate the feasibility, cost‑effectiveness, and clinical impact of such an approach, as well as to identify the patient populations most likely to benefit from POCUS‑based surveillance.

Limitations

This report has several limitations. As a single case, its findings are not generalizable and cannot establish causality or define optimal management strategies. The detection of the thrombus was incidental, and the exact timing of thrombus formation remains uncertain, particularly given prior imaging that did not report this finding. Additionally, POCUS is operator-dependent and its diagnostic accuracy for intracardiac thrombus may vary based on image acquisition and clinician experience. Finally, the absence of systematic screening limits the ability to determine the true prevalence of clinically silent right atrial thrombi in similar high-risk populations.

## Conclusions

This case demonstrates that bedside POCUS can identify clinically significant, otherwise unsuspected intracardiac pathology and directly influence patient management. In patients with risk factors such as indwelling catheters and hypercoagulable states, maintaining a low threshold for cardiac imaging may enhance early detection of high-risk intracardiac thrombi and potentially facilitate timely intervention in selected patients.
